# Geographical variation in the clinical profile of patients with *Candida auris*

**DOI:** 10.1017/ash.2025.10064

**Published:** 2025-07-28

**Authors:** Taylor Schieber, Kaleb Roemer, Kenneth Sands, Nickie Greer, Julia Moody, Troy Watson, Adam Hasse

**Affiliations:** HCA Healthcare, Nashville, TN, US

## Abstract

**Objective:**

To describe *Candida auris* infections from two different geographical regions within a large health-system, both of which have experienced a significant increase in the occurrence of *C. auris.*

**Design:**

Multicenter, retrospective, descriptive analysis across a large healthcare system.

**Methods:**

Patients were included in this study if they were admitted as an inpatient between January 1, 2021 and September 30, 2022 and had a clinical specimen that grew *C. auris*.

**Results:**

A total of 321 patients were included. The clinical outcomes of included patients were comparable between geographical regions (Western and Eastern), with the exception of patients who experienced mortality or transitioned to hospice care at discharge (Western 32.1% vs Eastern 19.1%, *P* = .014). Over one-third of patients required mechanical ventilation at any point during their admission, while greater than half of the total study population had receipt of a blood transfusion. Approximately 25.2% of all patients received hemodialysis, while 24.3% received total parental nutrition during their hospital stay. More than 50% of patients in both regions required an admission to the intensive care unit at any time-frame during their stay. Fluconazole-resistant isolates were more prevalent in the Western region, but both regions demonstrated a high prevalence of resistance.

**Conclusion:**

Patients identified with *C. auris* were characterized by significant underlying morbidity and disease burden. Further studies are warranted to identify infection prevention best practices to reduce transmission and reduce mortality through earlier identification and appropriate antifungal therapy.

## Introduction

*Candida auris* was first identified in 2009 in Japan, with the earliest known strain thought to have occurred in 1996 in South Korea.^1, 2^ Since its identification, it has emerged as a multi-drug resistant organism (MDRO) that presents a serious global threat.^1–7^ This specific species of yeast has gained significant attention over recent years due to major outbreaks of invasive infections in the healthcare setting and its unique resistance profile to current antifungal treatment options.^1–6^ In 2019, the Centers for Disease Control and Prevention (CDC) included *C. auris* as an urgent threat associated with increased morbidity and mortality rates in the *Antibiotic Resistance Threat Report*.^3^ Current literature suggests that *C. auris* infections are associated with significant morbidity and mortality, with mortality rates ranging between 30% and 72% in patients with invasive infections.^1,2,4,6,8,9^

*C. auris* differs from other *Candida* species due to several characteristics. It is not considered a resident commensal organism and is not typically present within the human gastrointestinal tract, unlike other *Candida* species. It has a unique ability to contaminate surfaces and medical devices within the hospital environment, despite the use of disinfectants, and remains viable for numerous months.^4–6^ This allows the transmissibility of the pathogen to be exceptionally high and rapid, resulting in an increased risk for prolonged outbreaks in healthcare facilities. Another distinctive characteristic of *C. auris* is its multi-drug resistant susceptibility profile. It is the only fungal species to demonstrate intrinsic resistance to the three main classes of antifungals: azoles, amphotericin B, and echinocandins.^7,8,10,11^ This makes it challenging when treating patients with *C. auris* infections, especially invasive infections. Although nearly all clinical cases of *C. auris* have shown some level of antifungal resistance, current literature does vary regarding the susceptibility patterns to specific agents.^7,8,10,11^ A CDC Morbidity and Mortality Report from 2020, which evaluated 331 *C. auris* isolates, found that over 99% were resistant to fluconazole, 61% were resistant to amphotericin B, and 4% were resistant to echinocandins.^10^ Susceptibility to antifungal agents also has been found to be dependent on the specific clade of *C. auris* that is identified, which is distinct by geographical region. Clades I-IV have all been identified within the United States.^12,13,14^

*C. auris* infection rates have continued to increase in the United States.^9,15,16,17^ Specifically within the studied healthcare system, geographical distribution of *C. auris* infections have primarily been in the Florida and Nevada regions. The healthcare system witnessed significant increases of cases in Nevada over a short period, while Florida had a more gradual increase in cases.^18^ Limited literature currently exists in regard to risk factors associated with *C. auris* infections, especially when evaluating the role geographical location plays in the acquisition of this pathogen. There have been many proposed risk factors that could potentially predispose patients to *C. auris* infections. Generally, these are similar to other *Candida* infections, such as prolonged intensive care unit (ICU) admission, total parenteral nutrition (TPN) administration, and the presence of indwelling devices.^1,2,8^ There is also limited data on the comorbid conditions associated with the pathogen. The objective of this study is to describe *C. auris* infections from two different geographical regions within a large health-system, both of which have experienced a significant increase in the occurrence of *C. auris,* but with distinct clades. Our aim was to describe patient factors associated with the organism and identify similarities and differences in relation to geographic location.

## Methods

We conducted a multicenter, retrospective, descriptive analysis within a large healthcare system of over 180 acute care facilities in 20 states across the United States. We identified patients if they had a positive clinical culture for *C. auris* from any source and at any time after hospitalization within the healthcare system. Thirty-seven hospitals across 5 states were identified to have at least one patient meeting study inclusion criteria. Patients were included in this study if they were admitted as an inpatient, at least 18 years old upon discharge from inpatient care, had an admission date between January 1, 2021 and September 30, 2022, and had a valid clinical specimen that grew *C. auris* documented in both the electronic health record (EHR) and Premier TheraDoc®, a clinical surveillance tool used to track hospital-acquired infections and other hospital-related safety issues. *C. auris* identification was by MALDI-TOF method. Positive cultures could potentially represent clinical cultures or colonization, but cultures or positive PCR swabs identified as surveillance or point prevalence screens were excluded. Facilities within the healthcare system were not performing routine admission screening for *C. auris* during the study period, but point prevalence surveillance was completed on all patients in the same hospital unit of an identified *C. auris* isolate at the guidance of state and local health departments. Patients were excluded if they were missing any required inpatient data, such as medication orders and administrations, specimen collection, and demographics. Data were collected from the EHR of patients admitted to facilities within the large healthcare system. EHR data collected during the course of patients’ clinical care within the healthcare system are input into an enterprise data warehouse in a standardized manner for analysis. This research was IRB exempt in accordance with institutional policy, as no sensitive patient-specific information was involved. This research was supported (in whole or in part) by HCA Healthcare and/or an HCA Healthcare affiliated entity. The views expressed in this publication represent those of the author(s) and do not necessarily represent the official views of HCA Healthcare or any of its affiliated entities.

The primary goal was to evaluate the clinical characteristics associated with patients with *C. auris* and identify variation based on geographical location. Secondary outcomes included antimicrobial utilization and exposure at any time during hospitalization, prevalence of concomitant MDROs, source of *C. auris* index culture, and subgroup analysis that included the following: patients that experienced mortality or transitioned to hospice care upon discharge, patients that required mechanical ventilation, patients who were admitted to the ICU at some point during their hospital admission, and patients with the presence of concomitant MDROs other than *C. auris*.

The Western Region included patients with a positive clinical culture for *C. auris* admitted to a facility within the healthcare system west of the Mississippi River. The Eastern Region included patients with a positive clinical culture for *C. auris* admitted to a facility within the healthcare system east of the Mississippi River. Eastern and Western regions were chosen for comparison based on evolving epidemiology data for different clades in different regions across the United States.^12^ Patient demographics, comorbidities, and clinical characteristics were taken from the EHR. The primary inpatient admission was defined as the patient encounter associated with the positive *C. auris* specimen. Previous inpatient admission within prior 90 days was determined by an inpatient admission at the same facility that has a discharge date and time within 90 days of the primary admission. Time to *C. auris* culture was defined as admission date to collection date of index *C. auris* culture. Clinical characteristics include a COVID-19 positive test, mechanical ventilation, transfusion of blood products, dialysis, and TPN administration. Elixhauser comorbidity index scores were calculated as a proxy for comorbidities. Additional culture data were acquired to determine the presence of concomitant MDROs. An MDRO was defined as one of the following: methicillin-resistant *Staphylococcus aureus* (MRSA), vancomycin-resistant *Enterococcus*, extended-spectrum beta-lactamase-producing organisms (ESBLs), multi-drug resistant (MDR) *Pseudomonas aeruginosa* (defined as testing either intermediate or resistant to at least one drug in at least three of the following six categories: extended-spectrum cephalosporin, fluoroquinolones, aminoglycosides, carbapenems, piperacillin/tazobactam and cefiderocol), carbapenem-resistant *P. aeruginosa*, and carbapenem-resistant *Acinetobacter* species. Antimicrobial exposure was defined as having receipt of at least one dose of the following agents at any point during hospitalization or within the previous 90 days: vancomycin, cefepime, ceftriaxone, meropenem, linezolid, piperacillin/tazobactam, metronidazole, fluoroquinolones, micafungin, and fluconazole.

Patient baseline and clinical characteristics were expressed with frequencies and percentages for categorical data and either medians with interquartile ranges or means with standard deviations for continuous data. Statistical analyses were completed in R Studio (version 2023.06.0). Elixhauser comorbidity index scores were calculated using the comorbidity package from ICD-10 codes.^19^ Multivariable logistic regression analysis was completed to evaluate mortality or discharge to hospice differences between variables. *P*-values for differences in proportions between the two groups were calculated via Pearson’s chi-squared test with a continuity correction, while *P*-values between group means and group medians were calculated by *t*-tests and Mood’s median tests, respectively. Fisher’s Exact Test was used to determine p-values for antifungal resistance differences between regions. Significance was considered to be a *P* value less than .05.

## Results

**Figure 1.**
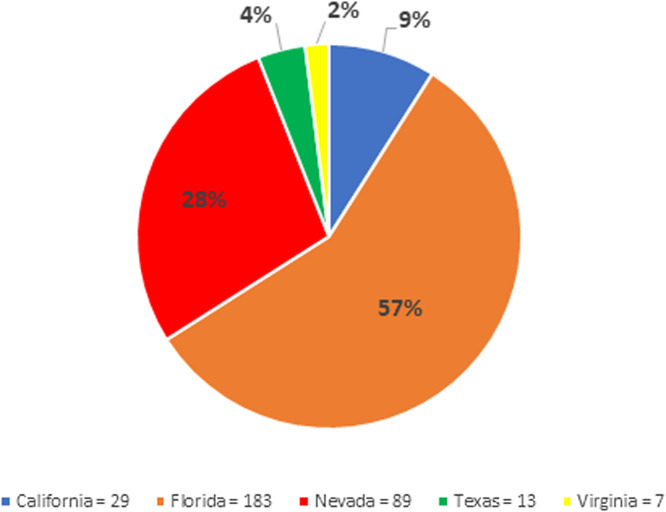
Pie chart of included patients based off location.

**Figure 2.**
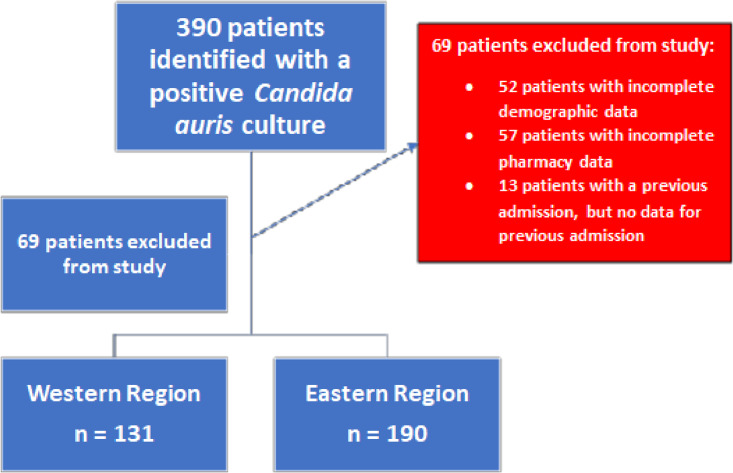
Flowchart of the patients included in the study.

**Figure 3.**
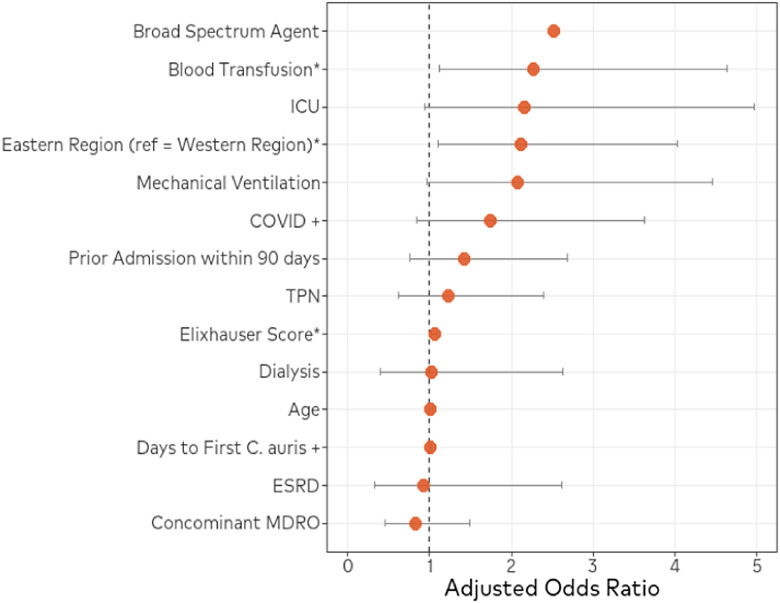
Multivariable logistic regression analysis for mortality and geographical region.

There were 390 unique patients with a positive culture for *C. auris* during the study time frame. From the identified patients, 69 patients were excluded from the study population for incomplete data, which left 321 patients included from 37 hospitals across 5 states (Figures 1 and 2). The Western Region contained 131 patients while the Eastern Region contained 190 patients. Hospitals in Nevada were responsible for the majority of patients in the Western Region, as they had 118 of the 131 patients, and hospitals across Florida were responsible for the majority of patients in the Eastern region, contributing 183 of the 190 patients. The average age for the Western and Eastern Region was 60.1 years and 64.4 years, respectively (*P* = .019). Median length of stay was 32 days and 20.5 days for the Western and Eastern Region, respectively (*P* = .002). All baseline characteristics are listed in Table 1. Over 60% of patients had index *C. auris* culture obtained after day 3 of hospital admission (Western 66.4% vs Eastern 56.8%). Patients were also likely to have a previous hospitalization within the prior 90 days in both the Western and Eastern regions (57.3% vs 61.6%, *P* = .508), and a large proportion were re-admitted within 30 days of discharge (Western 53.8% vs Eastern 58.1%, *P* = .502). Subjects who experienced mortality or transitioned to hospice care at discharge was significantly higher in the Eastern Region compared to the Western Region (32.1% vs 19.1%, *P* = .014). Multivariable logistic regression analysis confirmed significant mortality or discharge to hospice differences for patients in the Eastern Region compared to the Western Region (aOR 2.110; 95% CI 1.107, 4.022), receipt of blood transfusion (aOR 2.272; 95% CI 1.113, 4.639), and Elixhauser score (aOR 1.061; 95% CI 1.027, 1.095) (Figure 3, Table 2).

**Table 1.** Baseline and clinical characteristics of patients with a positive *Candida auris* cultures separated by geographical location

Characteristic	Western region (n = 131)	Eastern region (n = 190)	P value
Mean age, years (SD)	60.1 (18.3)	64.4 (14.3)	0.019
Mean length of stay, days (SD)	48.1 (53.9)	35.3 (40.7)	0.016
Median length of stay, days (IQR)	32.0 (15, 59.5)	20.5 (10, 48.8)	0.002
Days to first *Candida auris* culture, mean (SD)	23.2 (30.2)	15.0 (21.3)	0.005
Days to first *Candida auris* culture, median (IQR)	14.4 (0.66, 37.0)	5.4 (0.06, 23.9)	
Expired/hospice care	19.1% (25)	32.1% (61)	0.014
Admitted within prior 90 days	57.3% (75)	61.6% (117)	0.508
Re-admitted within 30 days of discharge	53.8% (57)	58.1% (75)	0.502
Clinical characteristics			
COVID-19 positive	11.5% (15)	18.9% (36)	0.099
Mechanical ventilation	35.1% (46)	41.6% (79)	0.293
Blood transfusions	55.7% (73)	56.3% (107)	1.0
Hemodialysis	26.0% (34)	24.7% (47)	0.908
Presence of ESRD on admission	18.3% (24)	18.9% (36)	1.0
Total parenteral nutrition	24.4% (32)	24.2% (46)	1.0
Presence of concomitant MDRO	43.5 (57)	44.7 (85)	0.918
Mean Elixhauser comorbidity index score	16.0	16.4	0.708
Hospitalization included intensive care	51.9% (68)	55.3% (105)	0.553
Receipt of broad-spectrum agent	93.1% (122)	96.3% (183)	0.304

ESRD, end-stage renal disease.

**Table 2.** Multivariable logistic regression analysis for mortality and geographical region

Variable	Adjusted odds ratio	95% Confidence Interval	p-value
(Intercept)	0.003	(0.000, 0.055)	<.001
Age (years)	1.009	(0.989, 1.029)	.382
Days to First C. auris Positive Test	1.005	(0.993, 1.018)	.410
Prior Admission within 90 days	1.428	(0.757, 2.691)	.271
COVID Positive	1.745	(0.841, 3.62)	.135
Mechanical Ventilation	2.076	(0.968, 4.453)	.061
Blood Transfusion	2.272	(1.113, 4.639)	.024
Dialysis	1.026	(0.4, 2.635)	.957
ESRD	0.932	(0.332, 2.614)	.893
TPN	1.225	(0.626, 2.398)	.554
Concomitant MDRO	0.825	(0.454, 1.497)	.526
Eastern Region (ref = Western Region)	2.110	(1.107, 4.022)	.023
Elixhauser Score	1.061	(1.027, 1.095)	<.001
ICU	2.162	(0.942, 4.963)	.069
Broad Spectrum Agent	2.522	(0.233, 27.25)	.446

The clinical characteristics of included patients were comparable between regions (Table 1). Over one-third of patients required mechanical ventilation at any point during their admission, while greater than half of the total study population had receipt of a blood transfusion. Over 25% of all patients received hemodialysis, while 24.3% received TPN during their hospital stay. There were no significant differences of hemodialysis or TPN between the regions. Almost half of the total study population had the presence of an MDRO during the same admission as the *C. auris* index culture, with methicillin-resistant *S. aureus* (MRSA) and MDR *P. aeruginosa* being the most common in the Western Region and ESBL-producing organisms and MDR *P. aeruginosa* the most common in Eastern Region. The average Elixhauser Comorbidity Index Score for the Western and Eastern regions was 16 and 16.4, respectively. The percentage of patients whose hospitalization required an admission to the ICU at any time-frame during their stay was more than 50% in both groups. Less than 10% were admitted to the ICU as their initial level of care. The most common admitting diagnosis was sepsis, representing 28.7% of patients. The top three index sources of *C. auris* cultures were blood (30.8%), skin/soft tissue (28.7%), and the urinary tract (22.7%).

Antimicrobial exposure was also similar between Western and Eastern Region patients, with vancomycin being the most common in both regions (Western 67.9% vs Eastern 74.7%). Many patients had receipt of antipseudomonal antibiotics during their admission as 60% of the total population received cefepime. For antifungal exposure, 72.5% and 69.5% had exposure to micafungin at some point during admission for the Western and Eastern regions. Fluconazole resistance was significantly different between the Western and Eastern regions (95.7% vs 86.0% resistance) (Table 3).

**Table 3.** Microbiology data

Source	*Candida auris* Isolated (*n* = 321)		
Blood	30.8% (99)		
Skin/soft tissue	28.7% (92)		
Urinary Tract System	22.7% (73)		
Respiratory/pulmonary	11.2% (36)		
Other	6.5% (21)		

Antifungal Resistance	Western Region (*n* = 131)	Eastern Region (*n* = 190)	*p*-value
Susceptibilities present	87.8% (115)	86.3% (164)	**-**
Amphotericin B Resistant	5.2% (6)	10.4% (17)	.184
Fluconazole Resistant	95.7% (110)	86.0% (141)	.008
Anidulafungin Resistant	0.0% (0)	0.6% (1)	1.000
Caspofungin Resistant	0.9% (1)	2.4% (4)	.652
Micafungin Resistant	0.0% (0)	0.6% (1)	1.000

Only index culture source and susceptibilities were captured and shown here. Percent resistant only includes those with susceptibility cultures. Resistance interpretation based on established guidelines provided by the Clinical and Laboratory Standard Institute and are as follows listed as tentative MIC breakpoints: amphotericin *B* ≥ 2 ( ≥ 1.5 if Etest is used), fluconazole ≥32 mcg/mL, anidulafungin ≥4, caspofungin ≥2, and micafungin ≥4.

Several subgroup analyses were conducted. Patients who experienced mortality or transitioned to hospice care had a significantly longer time to first positive *C. auris* compared to those who did not (17 d vs 5 d, *P* = .007). The clinical profile was significantly different for the mortality and hospice care subgroup except for the presence of concomitant MDROs (Table 4).

**Table 4.** Baseline and clinical characteristics of patients with a positive *Candida auris* culture who experience mortality or transitioned to hospice care vs those who did not

Characteristic	No Mortality/Hospice Care (*n* = 235)	Mortality/Hospice Care (*n* = 86)	*P* value
Mean age, years (SD)	62.1 (15.7)	63.9 (17.4)	.375
Mean length of stay, days (SD) Median length of stay, days (IQR)	39.3 (48.4) 23.0 (11, 50.5)	43.9 (42.4) 34.0 (12, 56.5)	.432 .072
Days to first *Candida auris* culture, mean (SD) Days to firs Candida auris culture, median (IQR)	16.1 (24.6) 5.0 (0.08, 23.8)	24.6 (27.2) 17.0 (1.4, 38.2)	.008
Admitted within prior 90 days	61.7% (145)	54.7% (47)	.569
Clinical characteristics COVID-19 positive Mechanical ventilation Blood transfusions Dialysis Total parenteral nutrition Presence of concomitant MDRO	11.5% (27) 27.7% (65) 48.1% (113) 21.7% (51) 20.4% (48) 42.1% **(**99)	27.9% (24) 69.8% (60) 77.9% (67) 34.9% (30) 34.9% (30) 50.0% (43)	**.001** **<.001** **<.001** **.024** **.011** **.258**
Mean Elixhauser comorbidity index score	14.2	21.8	**<.001**
Hospitalization included intensive care	43.0% (101)	83.7% (72)	**<.001**

## Discussion

This is the largest collection of clinical cases of *C. auris* in the United States to date. This study describes patient characteristics observed in a population with this specific fungal pathogen, and how they differ in association with geographical location. It is important to consider whether the clinical profile of patients with *C. auris* may be different based on geographical location, given the known variation in the specific clades of *C. auris* identified by region as well as potential differences in local epidemiology.^12^

In general, the data presented in this study suggests that *C. auris* is associated with patients at increased risk of mortality. High mortality rates among patients with *C. auris* have been reported in other studies as well.^1,2,8,9^ Specifically, the study conducted by Simon, et al demonstrated an in-hospital mortality rate of 44.6% in patients with *C. auris* candidemia.^1^ However, it was a small study that only included 83 patients. A study by Benedict et al analyzed a total of 192 *C. auris* hospitalizations across 42 hospitals. This study reported 34% of patients experienced in-hospital mortality or transitioned to hospice care, similar to the current study. The study by Benedict et al also reported similar clinical patient characteristics to our study, as 75.5% of the hospitalizations in their study involved an ICU stay and mechanical ventilation was used 43% of hospitalizations. Interestingly, a larger percentage of patients included in the study had their first positive *C. auris* culture within 2 days of admission (55.5% vs 39.3%).^9^

Our study also demonstrated that patients with this pathogen typically have an elevated comorbidity index, with patients included in this study having an average Elixhauser Comorbidity Index Score of 16.2. This would suggest that those with significant comorbidities represents the profile of patients susceptible to this condition. Other literature has also shown that affected patients typically present with significant comorbidities. Other studies have demonstrated that these patients commonly have high-risk health conditions such as diabetes mellitus, cardiovascular disease, kidney disease, lung disease, and immunodeficiency.^2,20^ Other patient and clinical factors shown in this study that were similar between the two geographical locations included extended length of stay, recent previous hospital admission, ICU admission, mechanical ventilation, and receipt of blood transfusions, hemodialysis, and TPN. The high prevalence of patients with blood transfusions, hemodialysis, and TPN may simply reflect the presence of indwelling devices and catheters, which have already been established risk factors for fungal infections.^1,2,8^

The source of *C. auris* infections and colonization most commonly arise from the skin, such as the axilla and groin areas. These areas provide the ideal environment for the organism to colonize, replicate, and persist. We observed that the majority of subjects had a blood or skin and soft tissue source, which correlates with other current literature.^1,2,20^ We also observed that it is common for patients infected or colonized with *C. auris* to also have MDROs present. This may be due to the level of acuity and length of stay many of the study population experienced, which generally placed them at increased risk of infections and receipt of broad-spectrum antimicrobials.^21^

One significant difference observed between the regions was the rate of patients who experienced death or transitioned to hospice care at discharge. Although it is difficult to draw a definite reason for this difference, there are some possible explanations. One explanation is the possibility of different *C. auris* clades affecting the two groups. Clade IV isolates have been shown to be associated with the highest mortality rates, followed by isolates from clades I, III, and II.^13^ Isolates from clade II generally are more drug-susceptible compared to the other clades.^12,13^ The difference in virulence factors may have contributed to the difference of mortality and hospice care seen in the current study. While the majority of cases in the USA have been Cade I^22^, Clade III has predominated in the Nevada region that makes up the majority of samples for the Western region in this analysis.^18^ However, we were unable to test for specific clades in this study.

While this study is the largest collection of clinical cases of *C. auris*, there are several important limitations that should be noted. First, the study is observational in nature. Second, the included patients could have been colonized with *C. auris*, which limits the generalizability of the study strictly to patients with true clinical infections. Third, we cannot infer causality for either morbidity or mortality in relation to presence of *C. auris* in this study. Additionally, antimicrobial utilization represented any administration during hospitalization rather than exposure preceding obtainment of clinical *C. auris* culture that may have selected for it. Lastly, results of this study represent the experience of a single healthcare system, although it is a system with an expansive geographical presence in the United States.

In conclusion, patients identified with *C. auris* are characterized by significant underlying morbidity and disease burden. Further research is warranted to understand the attributable morbidity and mortality in association with this organism, specifically in patients with documented clinical infections. More information regarding appropriate infection prevention measures against *C. auris* is also warranted and more pointed studies analyzing confirmed clinical cultures, such as fungemia, which would not be representative of colonization.
